# GnRH Stimulation Test in Precocious Puberty: Single Sample is Adequate for Diagnosis and Dose Adjustment

**DOI:** 10.4274/jcrpe.v3i1.03

**Published:** 2011-02-23

**Authors:** Nurgün Kandemir, Hüseyin Demirbilek, Zeynep Alev Özön, Nazlı Gönç, Ayfer Alikaşifoğlu

**Affiliations:** 1 Hacettepe University Medical School, Division of Pediatric Endocrinology, Ankara, Turkey; +90 312 305 11 24+90 312 312 18 09 dr_huseyin@hotmail.comHacettepe University Medical School, Division of Pediatric Endocrinology, Sıhhiye, Ankara, Turkey

**Keywords:** Central precocious puberty, follicle stimulating hormone, luteinizing hormone, GnRH analogue, hypotalamo-pituitary-gonadal axis, GnRH stimulation test

## Abstract

**Objective:** Gonadotropin stimulation test is the gold standard to document precocious puberty. However, the test is costly, time-consuming and uncomfortable. The aim of this study was to simplify the intravenous gonadotropin-releasing hormone (GnRH) stimulation test in the diagnosis of precocious puberty and in the assessment of pubertal suppression.

**Methods:** Data pertaining to 584 GnRH stimulation tests (314 testsfor diagnosis and 270 for assessment of pubertal suppression) were analyzed.

**Results:** Forty-minute post-injection samples had the greatest frequency of “peaking luteinizing hormone (LH)” (p<0.001) in the diagnostic tests when the cut-off value was taken as 5 IU/L for LH, 40th minute sample was found to have 98% sensitivity and 100% specificity in the diagnosis of precocious puberty, while the sensitivity and specificity of the 20th minute sample was 100% in the assessment of pubertal suppression.

**Conclusion:** LH level at the 40th minute post-injection in the diagnosis of central precocious puberty and at the 20th minute post-injection in the assessment of pubertal suppression is highly sensitive and specific. A single sample at these time points can be used in the diagnosis of early puberty and in the assessment of pubertal suppression.

**Conflict of interest:**None declared.

## INTRODUCTION

Several aspects of the clinical and laboratory diagnosis of central precocious puberty (CPP) need further evaluation. Gonadotropin-releasing hormone (GnRH) stimulation test is the gold standard to document premature activation of the hypothalamic-pituitary-gonadal axis in cases with clinical signs and symptoms of early puberty (1,2). Repeated blood sampling at different time points (5 to 8 times) is required to measure the levels of follicle-stimulating hormone (FSH) and luteinizing hormone (LH) both before and after administration of GnRH (3,4,5). The duration of the test is 90 to 120 minutes and 15 to 25 mL of blood is needed for 10 to 16 measurements. The procedure is costly, time-consuming and uncomfortable for patients. To avoid these problems, several attempts such as measurement of basal gonadotropin levels or subcutaneous leuprolide acetate test with a single sample have been made (2,6,7,8,9). None of these alternative tests have been standardized sufficiently or proven to be equal or superior to GnRH test as yet. In cases of precocious puberty, the GnRH test may also need to be repeated during treatment with GnRH analogue to assess the effectiveness of suppression and to adjust the dose of the analogue (10,11). 

In the current study, which aimed to investigate whether the testing procedure for diagnosis/assessment of CPP could be simplified without changing its validity, we evaluated the predictive values of FSH and LH at each time point during the GnRH test. 

## METHODS

**Diagnostic Group**

This group comprised 263 girls presenting with early pubertal signs. The age of onset as well as the tempo of puberty were extracted from patient records. Physical examination included body weight, height, and estimation of breast and pubic hair development according to the Tanner classification. Bone age was assessed using the Greulich-Pyle method (12).

Indications for GnRH stimulation test were: 

1. Onset of pubertal signs and growth acceleration before the age of 8 years.

2. Accelerated pubertal progression associated with advanced bone age after the age of 8 years.

**Group Under Treatment with GnRH Analogue**

Patients whose diagnosis of CPP was confirmed by basal ort ic GnRH stimulated peak LH were treated with GnRH analogue (leuprolide acetate) to suppress puberty. These patients constituted this group. All these patients were initially treated with an intramuscular injection of leuprolide acetate 3.75 mg every 28 days. In order to ascertain the adequacy of suppression of puberty in these patients, a repeat GnRH test was performed in the course of their treatment (re-test). Re-tests were done 3 weeks after the third dose of the GnRH analogue. In patients with inadequate suppression of puberty, the dose of the GnRH analogue was doubled (see below). 

The GnRH test was performed at 800-830 a.m. An intravenous (IV) cannula was inserted and blood samples were collected for basal FSH and LH. Following administration of a standard dose of 100 mg GnRH (Gonadorelin acetate, Ferring^®^), blood samples for FSH and LH were obtained at the 20^th^, 40^th^, 60^th^ and 90^th^ minutes. FSH and LH were measured using chemiluminescent microparticle immunoassay (ARCHITECH System, Abbott Laboratory Diagnostics, USA). The minimum detectable concentration was 0.07 IU/L for both FSH and LH. A cut-off value of stimulated LH of greater than or equal to 5 IU/L was considered diagnostic for pubertal response in patients with pubertal signs (13). In the course of GnRH analogue therapy, a cut-off value of less than 2 IU/L for LH in IV GnRH stimulation test was deemed to demonstrate adequate suppression of puberty (10).

**Statistical Analysis**

Stastistical analysis was carried out using Statistical Package for the Social Sciences, version 15.0 for Windows (SPSS Inc., Chicago, IL). The data are given as means±SD. Frequencies were compared using the x^2^-test. The Friedman test was used for repeated measures, Wilcoxon rank test and Mann-Whitney U test were used to compare means. A p value of less than 0.05 was considered to be statistically significant.

The diagnostic values of FSH, LH as well as LH/FSH ratio at different time points during GnRH test were evaluated using receiver operating characteristic (ROC) analysis. 

## RESULTS

**Diagnostic Group**

Three hundred and fourteen tests for the diagnosis of CPP were performed in 263 girls with signs of early puberty. The mean values for chronological age, height age and bone age were 7.9±1.5, 8.8±1.8 and 9.5±1.9 years, respectively. The mean body weight was 32.2±8.2 kg Pubertal development was compatible with Tanner stage 2 in 62.5%, and with stage 3 in 37.5% of patients.  

The mean LH level at the 40^th^ minute of the test (7.9±9.9 IU/L) was higher than the mean values at all other time points (p<0.001). The peak LH level coincided with the 40th minute sample in 71% of patients, and this occurrence was significantly more frequent at that compared to the other time points (p=0.03)Table 1. In 152 out of 314 (48.4%) tests, the peak LH was above the cut-off, and the test was diagnostic for CPP. Among these patients, in 149/152 (98%) of the tests, the 40^th^ minute sample was diagnostic for CPP with an LH level above 5 IU/L, even if it was not the peak. Also in all prepubertal patients (162 tests), the 40^th^ minute sample LH was below 5 IU/mL, even if LH did not peak at the 40^th^ minute.

The mean FSH level at the 60^th^ minute of the test was 12.1±11.3 IU/L, a value higher than those at all other time points (p<0.001). Additionally, at the 60^th^ minute of the tests, the highest frequency of peak FSH (42%) was obtained (Table 1).

**Figure 1:**
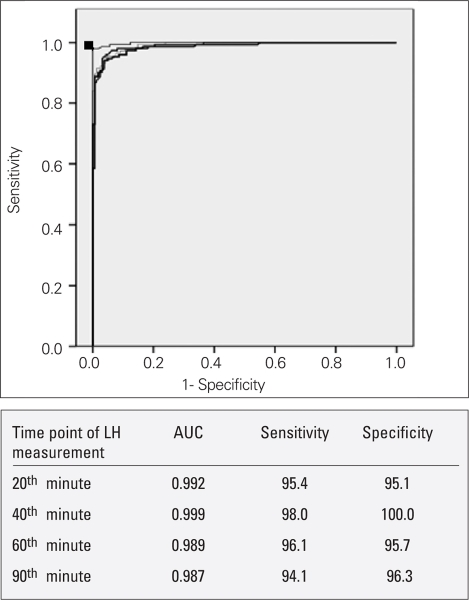
The ROC curves of LH at different time points of the test (20^th^, 40^th^, 60^th^ and 90^th^ minutes). LH: luteinizing hormone

Figure 1 presents the ROC curves of LH at different time points of the test. The ROC curve for LH at the 40^th^ minute was the most diagnostic one since it had the greatest area under the curve (AUC). 5 IU/L was considered as cut-off for LH in the diagnosis of CPP, and the ROC curves showed that a level of 4.93 IU/L at the 40^th^ minute was the most sensitive (98%) and specific (100%) cut-off for LH in the diagnosis.

The mean basal LH level in the group with pubertal response to the GnRH test was higher than that of the prepubertal group (1.05 and 0.22 mIU/L, respectively, p<0.0001). However, analysis of the diagnostic value of basal LH using ROC revealed that sensitivity and specificity of basal LH were both low (69.1% and 79.6%, respectively) in the diagnosis of CPP (Figure 2). Also, basal LH level in 13 of the 152 (8.5%) patients with a pubertal response to GnRH were below the lowest detectable level (<0.07 IU/L). When we considered basal LH ≥1 IU/L as the cut-off level for diagnosis of CPP, the positive predictive value of basal LH was 96.4% and the negative predictive value was 61.8%.                     

**Figure 3:**
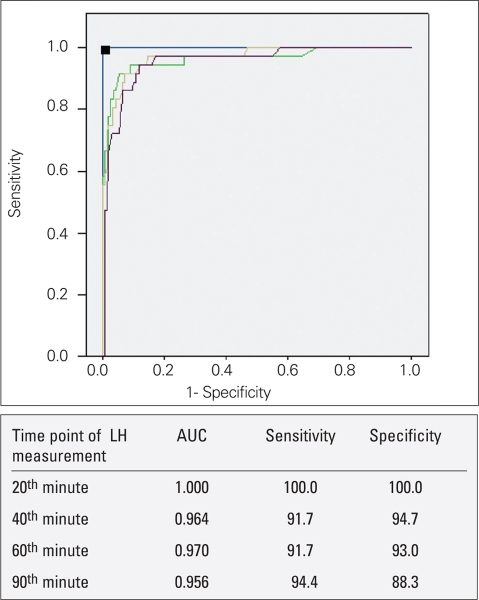
ROC curve of LH level at different time points of GnRH tests performed for assessment of pubertal suppression LH: luteinizing hormone, GnRH: gonadotropin-releasing hormone

Evaluation of LH/FSH ratio demonstrated no statistically significant difference in LH/FSH ratios between prepubertal and pubertal response groups both in the basal state (p=0.328) and in the peaks (p=0.718). Sensitivity and specificity of basal and peak LH levels are shown in Figure 3.

Evaluation of the diagnostic value of FSH using ROC analysis revealed that none of the time points, or any levels of FSH were sensitive or specific enough to allow a diagnosis of CPP.

**Group Under Treatment with GnRH Analogue**

Two hundred seventy tests were performed to assess pubertal suppression. The results are shown in Table 2. The mean LH level (1.2IU/L±0.9) was higher at the 20^th^ minute of the test in comparison to the other time points (p<0.001). Also, peak LH was noted at the 20^th^ minute in 249 of the 270 patients (92.2%). Two hundred and thirty-four patients (86.7%) were found to have a suppressed LH response to GnRH (peak LH < 2 IU/L). The peak LH level in 215 out of 234 patients (91.8%) with suppressed GnRH test was at the 20^th^ minute, whereas in the remaining 19 patients (8.2%), the peak LH level was at the 40^th^ minute.  In 36 (13.3%) patients, GnRH-stimulated LH peak was ≥2 IU/L, showing inadequate suppression, and the dose of the GnRH analogue had to be increased. The 20^th^ minute LH levels were ≥2 IU/L in all patients with inadequate suppression. Also, 20^th^ minute LH levels were <2 IU/L in all 234 patients (100%) with adequate suppression in response to GnRH test. In addition, the AUC in ROC analysis was greatest at the 20^th^ minute, with 100% sensitivity and specificity using a cut-off of 1.98 IU/L for LH. 

Evaluation of basal LH values for assessment of pubertal suppression using ROC analysis revealed that basal LH showed pubertal suppression with 83.3% sensitivity and 67.9%  specificity (AUC was 0.813 and LH cut-off value considered for pubertal suppression was 0.3 IU/L). According to this cut-off, the negative predictive value and the positive predictive value of basal LH were found to be 96.4% and 28.5%, respectively. 

**(Figure 2):**
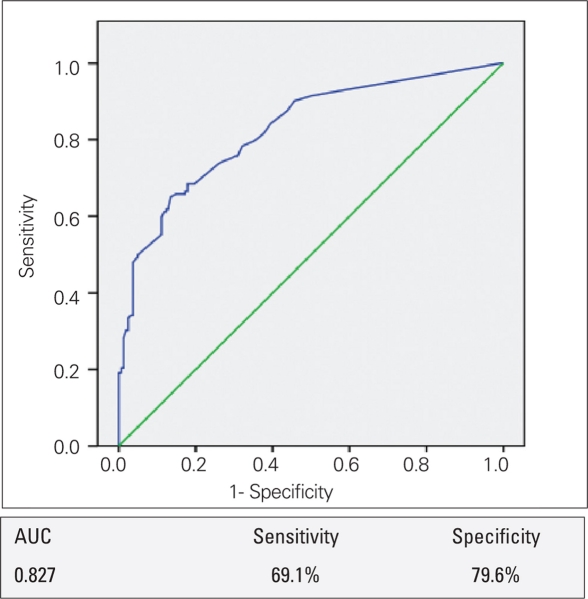
The ROC curve of basal LH levels in the diagnosis of CPP LH: luteinizing hormone, CPP: central precocious puberty

**Table 1:**
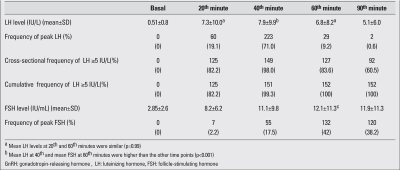
FSH, LH levels and frequency of peak LH and FSH at each time point during the GnRH stimulation test

**Table 2:**
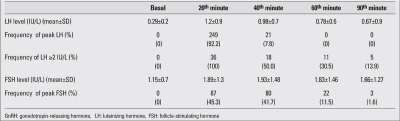
FSH, LH levels and frequency of peak LH at each time point during the GnRH stimulation test in patients under treatment with GnRH analogue

## DISCUSSION

GnRH test is the gold standard for the diagnosis of CPP. However, the test is time-consuming, costly and uncomfortable for the patients. In the current study, in an attempt to simplify the GnRH test, we analyzed the LH response to the GnRH test performed to diagnose precocious puberty and also to assess the adequacy of suppression with a GnRH analogue.  Different cut-off values of peak LH are used in the diagnosis of CPP in patients with early pubertal signs, and also in the assessment of pubertal suppression in patients under treatment with a GnRH analogue. Cut-off values also differ depending on the assay used to measure LH (5,13,14,15,16) In our clinic, LH is measured using ICMA, and LH ≥5 IU/L is considered to be diagnostic for CPP (13), whereas LH should be below 2 IU/L in order to consider a patient with CPP under GnRH analogue treatment as adequately suppressed (10). 

Comparison of peak LH levels at different time points during the GnRH test performed for the diagnosis of CPP showed that the mean LH value at the 40^th^ minute of the test was greater than those at other time points. Also, a greater number of LH peaks coincided with the 40^th^ minute samples. These findings suggest that a single sample obtained at the 40^th^ minute of the GnRH test may be used in the diagnosis of CPP with high sensitivity (98%) and specificity (100%).  

Previous studies reported LH to peak between 30 and 60 minutes of the GnRH test (5,13,14,17,18,19,20), but none of these studies aimed to simplify the standard IV GnRH test, except the study by Cavallo et al (5). These authors suggested that a single LH determination at the 30^th^ minute of the test was diagnostic. However, their study included only 55 diagnostic tests in 51 patients. To our best knowledge, the current study comprises the largest homogeneous population in this regard. 

Some authors suggest that LH/FSH ratio may also be valuable in the diagnosis of CPP (3,18,21). Jiang et al (18) reported that LH/FSH>0.9 at the 15th minute of the test might be diagnostic, but with lower sensitivity and specificity (80% and 90%, respectively). In another study, the peak LH/FSH >1 was found to have the highest positive predictive value (93.8%) (22). In the current study, both basal and stimulated LH/FSH ratios had lower sensitivity and specificity in comparison to the peak LH level in the diagnosis of CPP. Similarly, FSH levels, both basal and peak, had poor diagnostic value. Data from other studies also indicate that FSH has a poor diagnostic value in CPP (5).

In the last decade, the use of third-generation assays in the measurement of gonadotropin (FSH and LH) levels enabled clinicians to apply basal LH levels in the diagnosis of CPP (6,7). Neely et al (6) reported that a basal LH >0.1 IU/L was diagnostic for CPP with 94% sensitivity and 88% specificity. They also showed that a cut-off value >0.3 IU/L increased the specificity to 100% even though the sensitivity was reduced. Recently, Houk et al (7) suggested that using a basal LH >0.83 IU/L as the cut-off value yielded high sensitivity (93%) and specificity (100%) for the diagnosis of CPP. However, their study included a limited number of patients and the pubertal stage of the patients was not reported. According to our ROC curve analysis, basal LH > 0.3 IU/L had the highest sensitivitiy 65.1%) and specificity (86.4%). However, these values were lower than those reported in the study by Houk et al (7). When we considered basal LH>0.83 as the cut-off value, the sensitivity was found very low (34.2%), while the specificity was 96.3%. Furthermore, it was observed that 13 of the 152 patients (8.5%) diagnosed with CPP in the IV GnRH stimulation test had undetectable basal LH levels. When LH ≥1 IU/L was considered as the cut-off for the diagnosis of CPP, the positive predictive value of basal LH was found adequate to confirm the diagnosis of CPP without performing an IV GnRH stimulation test. Therefore, we do not suggest using basal LH as a single diagnostic criteria for CPP, except for LH ≥1 IU/L, which displayed a high positive predictive value (96.4%).

Analysis of pubertal suppression in 270 patients under treatment with a GnRH analogue for CPP revealed that LH level at the 20th minute of the test was 100% sensitive and specific to determine adequacy of suppression. Previous studies analyzed data regarding both diagnostic and follow-up GnRH tests; however, the specific question about the value of a single LH level to show the adequacy of pubertal suppression was not addressed (13). Cavallo et al (5) analyzed 39 tests in the course of treatment, but, their analysis was inconclusive owing to the small number of cases who were inadequately supressed.  Furthermore, because of its low sensitivity and specificity, we do not recommend basal LH value as a single criterion in the assessment of pubertal supression, with the exception of LH <0.3 IU/L, which displayed a high negative predictive value (96.4%). Therefore, basal LH can be used as a screening test to show pubertal suppression, and in case of LH ≥0.3, an IV GnRH stimulation test should be performed for dose adjustment. This cut-off value was previously presented as a criterion to differentiate prepubertal from pubertal patients  (6,13).

Separate analysis of the GnRH test in the diagnostic and treatment groups revealed an interesting finding. Gonadotropin levels peaked more rapidly in patients under treatment in comparison to those who were assessed for suppression. FSH levels also peaked more rapidly in the re-test group. 

In conclusion, a single sample of LH obtained at the 40^th^ minute of the GnRH test may be adequate for the diagnosis of CPP if a cut-off value of 5 IU/L is applied. Also, our findings indicate that a single measurement of LH at the 20^th^ minute of the GnRH test using a cut-off value of 2 IU/L is as valid as the standard test to evaluate pubertal suppression in patients under treatment with a GnRH analogue. 
